# A multisite evaluation of antifungal use in critical care: implications for antifungal stewardship

**DOI:** 10.1093/jacamr/dlac055

**Published:** 2022-06-23

**Authors:** C Logan, C Hemsley, A Fife, J Edgeworth, A Mazzella, P Wade, A Goodman, P Hopkins, D Wyncoll, J Ball, T Planche, S Schelenz, T Bicanic

**Affiliations:** Clinical Infection Group, St George’s University Hospitals NHS Foundation Trust, London, UK; Institute of Infection & Immunity, St George’s University of London, London, UK; Department of Infectious Diseases, Guy’s & St Thomas’ NHS Foundation Trust, London, UK; Infection Sciences, King’s College Hospital NHS Foundation Trust, London, UK; Department of Infectious Diseases, Guy’s & St Thomas’ NHS Foundation Trust, London, UK; Centre for Clinical Infection and Diagnostics Research, Department of Infectious Diseases, King’s College London Guy’s & St Thomas’ NHS Foundation Trust, London, UK; Clinical Infection Group, St George’s University Hospitals NHS Foundation Trust, London, UK; Institute of Infection & Immunity, St George’s University of London, London, UK; Department of Infectious Diseases, Guy’s & St Thomas’ NHS Foundation Trust, London, UK; Directorate of Pharmacy & Medicines Optimisation, Guy’s & St Thomas’s NHS Foundation Trust, London, UK; Department of Infectious Diseases, Guy’s & St Thomas’ NHS Foundation Trust, London, UK; Centre for Clinical Infection and Diagnostics Research, Department of Infectious Diseases, King’s College London Guy’s & St Thomas’ NHS Foundation Trust, London, UK; MRC Clinical Trials Unit at University College London, London, UK; Department of Critical Care, King’s College Hospital NHS Foundation Trust, London, UK; Department of Critical Care, Guy’s & St Thomas’ NHS Foundation Trust, London, UK; Department of Critical Care, St George’s University Hospitals NHS Foundation Trust, London, UK; Clinical Infection Group, St George’s University Hospitals NHS Foundation Trust, London, UK; Institute of Infection & Immunity, St George’s University of London, London, UK; Infection Sciences, King’s College Hospital NHS Foundation Trust, London, UK; Clinical Infection Group, St George’s University Hospitals NHS Foundation Trust, London, UK; Institute of Infection & Immunity, St George’s University of London, London, UK

## Abstract

**Background:**

ICUs are settings of high antifungal consumption. There are few data on prescribing practices in ICUs to guide antifungal stewardship implementation in this setting.

**Methods:**

An antifungal therapy (AFT) service evaluation (15 May–19 November 2019) across ICUs at three London hospitals, evaluating consumption, prescribing rationale, post-prescription review, de-escalation and final invasive fungal infection (IFI) diagnostic classification.

**Results:**

Overall, 6.4% of ICU admissions (305/4781) received AFT, accounting for 11.41 days of therapy/100 occupied bed days (DOT/100 OBD). The dominant prescribing mode was empirical (41% of consumption), followed by targeted (22%), prophylaxis (18%), pre-emptive (12%) and non-invasive (7%). Echinocandins were the most commonly prescribed drug class (4.59 DOT/100 OBD). In total, 217 patients received AFT for suspected or confirmed IFI; 12%, 10% and 23% were classified as possible, probable or proven IFI, respectively. Hence, in 55%, IFI was unlikely. Proven IFI (*n *= 50) was mostly invasive candidiasis (92%), of which 48% had been initiated on AFT empirically before yeast identification. Where on-site (1 → 3)-β-d-glucan (BDG) testing was available (1 day turnaround), in those with suspected but unproven invasive candidiasis, median (IQR) AFT duration was 10 (7–15) days with a positive BDG (≥80 pg/mL) versus 8 (5–9) days with a negative BDG (<80 pg/mL). Post-prescription review occurred in 79% of prescribing episodes (median time to review 1 [0–3] day). Where suspected IFI was not confirmed, 38% episodes were stopped and 4% de-escalated within 5 days.

**Conclusions:**

Achieving a better balance between promptly treating IFI patients and avoiding inappropriate antifungal prescribing in the ICU requires timely post-prescription review by specialist multidisciplinary teams and improved, evidence-based-risk prescribing strategies incorporating rapid diagnostics to guide AFT start and stop decisions.

## Introduction

Intensive care patients are at increased risk of invasive fungal infection (IFI) and consequently the ICU is a setting with high antifungal consumption.^1^ Invasive candidiasis (IC) is the most common IFI in the ICU, with invasive pulmonary aspergillosis (IPA) increasingly recognized. Both IC and IPA are associated with high crude mortality (∼40%–55%^2–4^ and ∼50%–80%^5,6^ respectively), with worse outcomes if treatment is delayed.^7,8^ Diagnosis is notoriously difficult; symptoms are non-specific, and conventional culture-based diagnostics have suboptimal sensitivity and prolonged turnaround time (TAT).^9,10^ Thus, recognizing the optimal timepoints to commence antifungal therapy (AFT) and identifying when antifungals can safely be stopped or de-escalated are challenges in the ICU.

Excessive antifungal use may cause individual and collective harm due to treatment-related adverse effects, drug–drug interactions and the selection of drug-resistant fungi, with increasing prevalence of fluconazole-resistant *Candida* (e.g. *Candida glabrata* and *Candida krusei)* and triazole-resistant *Aspergillus* (*Aspergillus fumigatus)* reported.^11,12^ Echinocandin-resistant *Candida* remains rare in the UK,^13^ but rising incidence is reported in the USA, associated with prolonged antifungal exposure.^14,15^ Furthermore, outbreaks of MDR *Candida auris* in ICUs globally have highlighted the detrimental clinical, operational and financial impacts of antifungal resistance.^16,17^

Optimizing antifungal use to attain the best clinical outcomes for patients, while preventing resistance emergence and minimizing drug toxicity, are key aims of antifungal stewardship (AFS) programmes.^18^ Challenges to AFS in the ICU include critical illness severity potentially driving overuse and hindering de-escalation, and organ dysfunction or drug–drug interactions restricting prescribing options from the limited antifungal classes available (azoles, echinocandins and polyenes).^19^ Minimizing inappropriate use of antifungal drugs is a priority in UK NHS hospitals, with a review of consumption included in the NHS England Medicines Optimisation Commissioning for Quality and Innovation (CQUIN) framework 2019–20.^20^ Information on antifungal prescribing practice and fungal epidemiology in the ICU is essential to provide a baseline from which stewardship initiatives can be developed, however data in this area are scarce. To this end, we undertook an evaluation of antifungal prescribing and IFI incidence in three critical care units in London teaching hospitals, to guide the development of local stewardship initiatives in the ICU.

## Methods

Between 15 May and 19 November 2019, each site undertook a service evaluation of antifungal prescribing on general surgical and medical ICUs at three tertiary London hospitals (St George’s University Hospitals NHS Foundation Trust [SGH], King’s College Hospital NHS Foundation Trust [KCH], and Guy’s & St Thomas’ NHS Foundation Trust [GSTT]) comprising over 165 total ICU beds covering a range of specialist services including trauma, haemato-oncology, bone marrow, renal and pancreatic transplant, and ECMO. All Trusts have ICU-specific antibiotic formularies, antimicrobial stewardship programmes and dedicated microbiology/ICU ward rounds on weekdays. All have on-site laboratories and use MALDI-TOF for fungal identification. The (1* *→* *3)-β-d-glucan (BDG) Fungitell assay (Associates of Cape Cod, Falmouth, MA, USA) and Bio-Rad Platelia *Aspergillus* galactomannan (GM) antigen sandwich enzyme immunoassay are used by all sites, however only KCH has on-site facilities for testing (at SGH and GSTT BDG and GM testing is outsourced); yeast susceptibility testing is performed on site at GSTT (Vitek 2/Sensititre YeastOne), but outsourced at KCH and SGH, and all sites outsource mould susceptibility testing.

All antifungal prescribing episodes were identified prospectively from electronic and pharmacy reporting records over the evaluation period. To assess consumption, antifungal drugs were classified into: (i) fluconazole; (ii) mould-active azoles (itraconazole, posaconazole, voriconazole, isavuconazole); (iii) echinocandins (anidulafungin, micafungin, caspofungin); (iv) amphotericin (liposomal and non-liposomal formulations); and (v) flucytosine. Timing, duration, clinical indication and rationale for prescribing were extracted from patient records. Antifungal consumption was defined as antifungal administered while on the ICU using days of AFT (DOT) per 100 occupied bed days (OBD); this measure allows for comparison between drugs and is a recommended antimicrobial utilization metric.^21^ ‘Rationale’ was defined as one of the following: prophylaxis; non-invasive infection; empirical; pre-emptive; or targeted (Table 1). We refer to empirical, pre-emptive and targeted rationales collectively as ‘prescribing for suspected or confirmed IFI’. Prescribing episode and duration were defined as the total number of DOT prescribed for a specific rationale, e.g. a patient on prophylactic AFT switched to another drug due to clinical deterioration and suspected IFI would have a prophylactic prescribing episode and an empirical episode.

**Table 1.** Definition of antifungal prescribing rationales

Rationale	Definition
Prophylaxis	AFT prescribed to prevent fungal infection
Non-invasive infection	AFT prescribed to treat superficial mucosal/skin infection/colonization
Empirical^a^	AFT prescribed in response to signs and symptoms of infection in an at-risk ICU host
Pre-emptive^a^	AFT prescribed in response to positive fungal biomarkers or radiology
Targeted^a^	AFT prescribed in response to microbiological evidence of proven IFI

a Empirical, pre-emptive and targeted prescribing defined collectively as ‘prescribing for suspected or confirmed IFI’.

To retrospectively evaluate appropriateness of AFT, the final IFI diagnostic classification for all patients prescribed AFT for suspected or confirmed IFI was assessed and classified as proven, probable or possible IFI or IFI unlikely (as defined in Table S1, available as Supplementary data at *JAC-AMR* Online^5,22^) considering host risk factors, radiological and microbiological investigations. Length of ICU and hospital stay and in-patient mortality were recorded to define outcomes.

For all empirical, pre-emptive and targeted AFT prescribing episodes for suspected or confirmed IFI, we recorded the timing and outcome of investigations sent and the timing and advice given in any documented post-prescription reviews by the microbiology/stewardship team. For AFT episodes initiated for suspected IFI but where IFI was ultimately not proven, we recorded whether antifungal de-escalation occurred, defined in this evaluation as either (i) AFT discontinuation or (ii) replacement of a broad-spectrum antifungal with a narrower-spectrum antifungal drug, within 5 days post-AFT initiation, similar to definitions used in other published antifungal de-escalation studies.^23,24^

To assess impact of BDG result on duration of prescribing in suspected but unproven IC, DOT per prescribing episode in those with a negative BDG, positive BDG and no BDG sent were compared. TAT was defined as the number of days between date of receipt of specimen in the lab to date of result authorization. For this evaluation, a BDG result of ≥ 80 pg/mL was considered positive, and <80 pg/mL negative. Only BDG results sent within 5 days prior to, or during, the prescribing episode were included. Episodes of AFT prescribed for proven IFI or suspected IPA were excluded from the analysis. Multivariable linear regression was used to model the number of DOT depending on the three BDG groups and BDG testing location (on-site testing [KCH], off-site testing [SGH and GSTT]).

For comparison of binary outcomes across categorical variables, univariable analysis was performed using χ^2^ or Fisher’s exact test. Analyses were performed using Graph Pad Prism (v9) and R (v 4.1.1). As this evaluation used routinely collected anonymized data collected by teams at each site to inform local development of AFS, it met the Health Research Authority (HRA) definition of a service evaluation and was registered and approved by the local audit and service evaluation governance bodies at each site.

## Results

There were 4781 patient admissions, accounting for 25* *040 OBD: 305 patients (6.4% of all admissions) received ≥1 antifungal drug(s) during their stay, accounting for 371 antifungal prescribing episodes and 2858 DOT (11.41 DOT/100 OBD) (overview in Table 2).

**Table 2.** Overview of antifungal prescribing episodes, rationale and duration

Variable	*n*
AFT prescribing episodes	371
Patients receiving AFT	305
Patients receiving ≥2 AFT episodes, *n* (%)	43 (14)
AFT episodes by prescribing rationale, *n* (%)	
Prophylaxis	69 (19)
Non-invasive	43 (12)
Empirical	183 (49)
Pre-emptive	31 (8)
Targeted	45 (12)
AFT duration by prescribing rationale (days), median (IQR)	
Prophylaxis	11 (5–23)
Non-invasive	5 (2–7)
Empirical	7 (3–10)
Pre-emptive	12 (7–22)
Targeted	15 (10–23)

### Antifungal consumption in ICU

**Figure 1.**
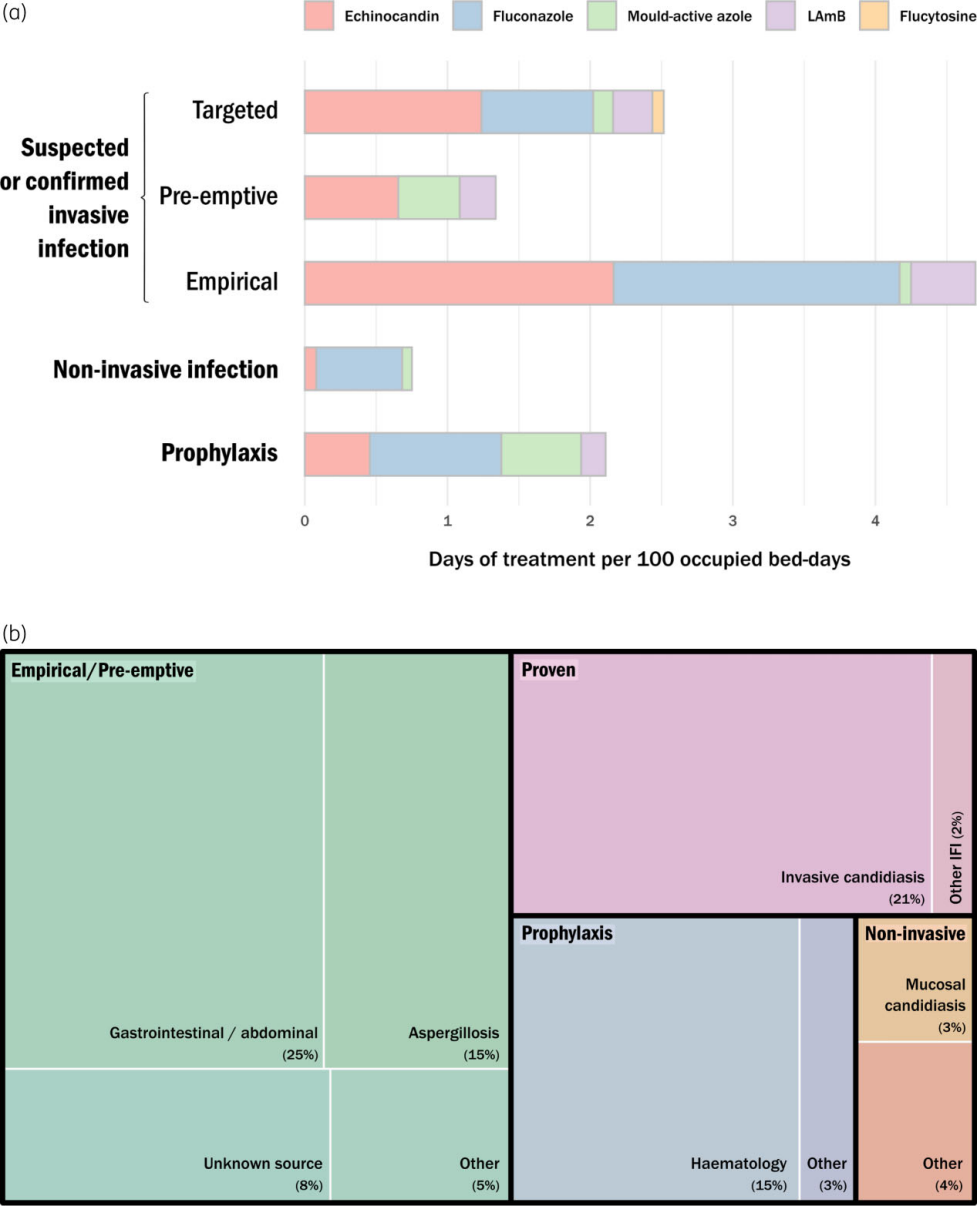
(a) Antifungal consumption by drug class according to rationale (prophylactic, non-invasive, empirical, pre-emptive and targeted therapy) in DOT/100 OBD. LAmB, liposomal amphotericin B. (b) Proportion of antifungal consumption (%) by prescribing indication. The area of each box is proportional to antifungal consumption for the prescribing indication in DOT/100 OBD (%). *Empirical/pre-emptive*: abdominal/gastrointestinal = 2.86 (25%); suspected invasive aspergillosis = 1.67 (15%); unknown source = 0.93 (8%); other = 0.52 (5%); *Proven*: invasive candidiasis = 2.37 (21%); other = 0.24 (2%). *Prophylaxis*: haematological = 1.76 (15%); other = 0.34 (3%); *Non-invasive infection/colonization:* mucosal candidiasis = 0.32 (3%); other = 0.41 (4%).

Antifungal prescribing for suspected or confirmed IFI accounted for 75% of consumption in critical care and was predominantly empirical (empirical 41%, 4.70 DOT/100 OBD; pre-emptive 12%, 1.34 DOT/100 OBD; targeted 22%, 2.51 DOT/100 OBD), with a further 18% used for prophylaxis (2.11 DOT/100 OBD) and 7% for non-invasive infection (0.75 DOT/100 OBD) (Table 3 and Figure 1a). Consumption by prescribing indication is illustrated in Figure 1(b). Empirical antifungal prescribing for a suspected abdominal/GI focus of IFI accounted for a quarter of all ICU antifungal consumption (25%, 2.86 DOT/100 OBD). Treatment for suspected IPA accounted for 15% (1.67 DOT/100 OBD) and proven IC 21% (2.37 DOT/100 OBD) of consumption given the longer treatment durations. For prophylactic antifungal use, underlying haemato-oncology disease was the most common indication and accounted for 15% (1.76 DOT/100 OBD) of all ICU consumption. When analysing by drug class, consumption was dominated by echinocandins (40%, 4.59 DOT/100 OBD) and fluconazole (38%, 4.31 DOT/100 OBD). For empirical therapy, similar proportions of fluconazole (43%) and echinocandins were used (46%), with minimal mould-active azole (2%) or amphotericin (10%) use (Figure 1a). Echinocandins accounted for a greater proportion of targeted prescribing (49%), whereas pre-emptive prescribing was dominated by mould-active azoles (32%) and amphotericin (18%), for suspected IPA. Prophylaxis was dominated by fluconazole (44%) and mould-active azoles (27%) largely for haemato-oncology patients on ICU, and the majority of non-invasive prescribing was fluconazole (80%). A fifth of patients prescribed antifungals during their ICU stay received ≥2 drug classes (21%, *n *= 64), and combination therapy was used in 3% (*n *= 9) of patients. An antifungal drug was stopped or switched to an alternative agent due to toxicity in 5% (17/371) of prescribing episodes; implicated drugs were fluconazole (*n *= 5); mould-active azoles (*n *= 5); amphotericin (*n *= 5); and echinocandins (*n *= 2), with the most common reasons being hepatoxicity (*n *= 8), cardiotoxicity (*n *= 3), renal toxicity (*n *= 2), electrolyte disturbance (*n *= 2) and hypersensitivity reactions (*n *= 2).

**Table 3.** Overview of antifungal consumption by drug class and prescribing rationale

Antifungal consumption	DOT, *n* (%)	DOT/100 OBD
Total	2858	11.41
Consumption by drug class		
Echinocandin	1150 (40)	4.59
Fluconazole	1080 (38)	4.31
Mould-active azole	320 (11)	1.28
Amphotericin	288 (10)	1.15
Flucytosine	20 (<1)	0.08
Consumption by rationale		
Prophylaxis	528 (18)	2.11
Non-invasive	188 (7)	0.75
Empirical	1177 (41)	4.70
Pre-emptive	335 (12)	1.34
Targeted	630 (22)	2.51

### Antifungal prescribing for suspected or confirmed IFI (empirical, pre-emptive and targeted)

To retrospectively assess how appropriately AFT was targeted in the ICU, the ultimate diagnostic classification of those prescribed AFT for suspected or confirmed IFI (all empirical, pre-emptive or targeted prescribing) was assessed. In 217 adults (median age 56 years, 72% male) there were 258 AFT prescribing episodes (Table 4). Blood culture, BDG, serum and BAL GM were sent in 88%, 36%, 19% and 9% of episodes, respectively. Eighty-five percent of episodes were in at-risk hosts with a median of 3 (IQR 2–4) of the following IFI risk factors: intubation, RRT, ECMO, total parental nutrition, central vascular catheter, surgery, solid organ transplant, haematological or other malignancy, or immunosuppressive therapy.

**Table 4.** Demographics, risk factors, and diagnostic classification for patients prescribed AFT for suspected or confirmed IFI (all empirical, pre-emptive, targeted prescribing)

Variable	*n*
Patients receiving AFT for suspected or confirmed IFI	217
Age in years, median (range)	56 (18–92)
Male, *n* (%)	156 (72)
Type of ICU admission, *n* (%)	
Surgical/trauma	132 (61)
Medical	85 (39)
Risk factor for IFI, *n* (%)	
Antibiotic therapy^a^	206 (97)
Central vascular catheter	180 (83)
Mechanical ventilation	152 (70)
Surgical procedure^b^	102 (47)
Steroids^c^	45 (21)
Renal replacement therapy	72 (33)
Immunosuppressive therapy^d^	45 (21)
Total parenteral nutrition	48 (22)
Diabetes mellitus	43 (20)
Haematological malignancy/BMT^e^	36 (17)
Malignancy (other)	33 (15)
Extracorporeal membrane oxygenation	27 (12)
Neutropenia	21 (10)
Solid organ transplant	3 (1)
*Candida* colonization^f^	
* Candida* colonization ≥1 site	107 (49)
* Candida* colonization ≥2 sites	38 (18)
IFI diagnostic classification^g^	
Proven IFI	50 (23)
IC	46
Other yeast	2
Invasive mould infection	2
Probable IFI	22 (10)
Probable IC	12
Probable IPA	10
Possible IFI	25 (12)
Possible IC	14
Possible IPA	11
Total proven/probable/possible IFI	97 (45)
IFI unlikely	120 (55)

BMT, bone marrow transplant.

a Within past 14 days.

b Surgical procedure; abdominal/upper GI, *n* = 73; cardiothoracic, *n* = 18; urological, *n* = 5; other, *n* = 6.

c Within past 1 month, ≥40 mg prednisolone or equivalent per day.

d Within past 3 months, including chemotherapy, monoclonal antibodies, Mycophenolate Mofetil, calcineurin inhibitors, cyclophosphamide.

e In total, 11/36 had undergone bone marrow transplant.

f In total, 207/217 had ≥1 sites sampled, and 192/217 had ≥2 sites sampled.

g Proven IFI: IC; candidaemia *n *= 27, deep-seated candidiasis without candidaemia *n *= 19 with *C. albicans* (61%, *n *= 28) being the most commonly isolated species, followed by *C. glabrata* (17%, *n *= 8), *C. parapsilosis* (11%, *n *= 5), *C. auris* (4%, *n *= 2), *C. tropicalis* (2%, *n *= 1), *C. dubliniensis* (2%, *n *= 1) and mixed (*C. albicans/C. glabrata/C. dubliniensis*, 2%, *n *= 1). Other yeast: pneumocystis jirovecii pneumonia (PJP), *n *= 1 (included as on empirical antifungal) and *Saccharomyces cerevisiae* (blood culture), *n *= 1. Invasive mould infection: *Scedosporidium apiospermum* (CSF and blood culture) *n *= 1 and *Scedosporidium prolificans* (blood culture) *n *= 1.

The IFI diagnostic classification was proven (IC *n *= 46; invasive mould *n *= 2; other yeast *n *= 2), probable (IPA  *n *=10, IC *n *= 12) or possible IFI (IPA *n *= 11, IC *n *= 14) in 45% (Table 4). Thus in 55% of patients prescribed AFT for suspected infection, IFI was unlikely. Hence, of all ICU admissions (*n *= 4781), 1% were admitted with (*n *= 6) or developed (*n *= 44) proven IFI, and a further 1% (*n *= 47) had probable or possible infection.

In proven infection the median time from ICU admission to diagnosis was 9 days (IQR 1–13). Proven infection was predominantly due to IC (*n *= 46, 92%; 27 candidaemia, 19 deep-seated candidiasis). Excluding those admitted with IC, the overall IC incidence in ICU was 8.5 cases per 1000 admissions (candidaemia and deep-seated candidiasis; 4.6 and 3.9 per 1000 admissions, respectively). The source was abdominal/GI in half of all proven IC (50%, *n *= 23), followed by vascular-catheter related (17%, *n *= 8), renal tract (9%, *n *= 4), cardiothoracic (4%, *n *= 2), endocarditis (4%, *n *= 2) and other (*n *= 7, Table 4). *Candida albicans* (61%, *n *= 28) was the most common cause of proven IC, followed by *C. glabrata* (17%, *n *= 8), *Candida parapsilosis* (11%, *n *= 5), *C. auris* (4%, *n *= 2), *Candida tropicalis* (2%, *n *= 1), and *Candida dubliniensis* (2%, *n *= 1). Proven mould infection (*n *= 2) and infection with other invasive yeasts (*n *= 2) were rare and occurred in immunocompromised hosts. Of those with proven IFI, 52% (*n *= 26) had a BDG sent; the result was positive (≥ 80 pg/mL) in 77% (*n *= 20), however the result was only available prior to diagnostic culture in three cases.

In IC, a significantly lower proportion of those with candidaemia had been empirically initiated on antifungal treatment prior to microbiological diagnosis (i.e. yeast identification in blood/sterile site culture) compared with those with *Candida* isolated from a non-blood sterile site (37% [*n *= 10] versus 74% [*n *= 14], *P *= 0.02). Hence, overall for IC 48% (*n *= 24) were on treatment prior to microbiological diagnosis.

Compared with patients with no IFI, those with proven/probable/possible IFI had a significantly greater length of ICU stay (15 versus 23 days, *P *= 0.01) and hospital stay (37 versus 55 days, *P *= 0.01), however there was no difference in crude in-hospital mortality (40% in both groups, *P *= 0.99).

### Post-prescription review and antifungal de-escalation

Review and de-escalation of all prescribing episodes for suspected or proven IFI were evaluated (*n *= 258). 79% (*n *= 204) of prescribing episodes had a documented review by members of the infection/microbiology team, with a median time to review of 1 day (IQR 0–3). The most common advice was to send investigations (58%, *n *= 121), recommend a duration of therapy (40%, *n *= 83), to stop antifungal treatment (38%, *n *= 79), to start AFT (38%, *n *= 79), or to switch to an alternative drug (19%, *n *= 40).

**Figure 2.**
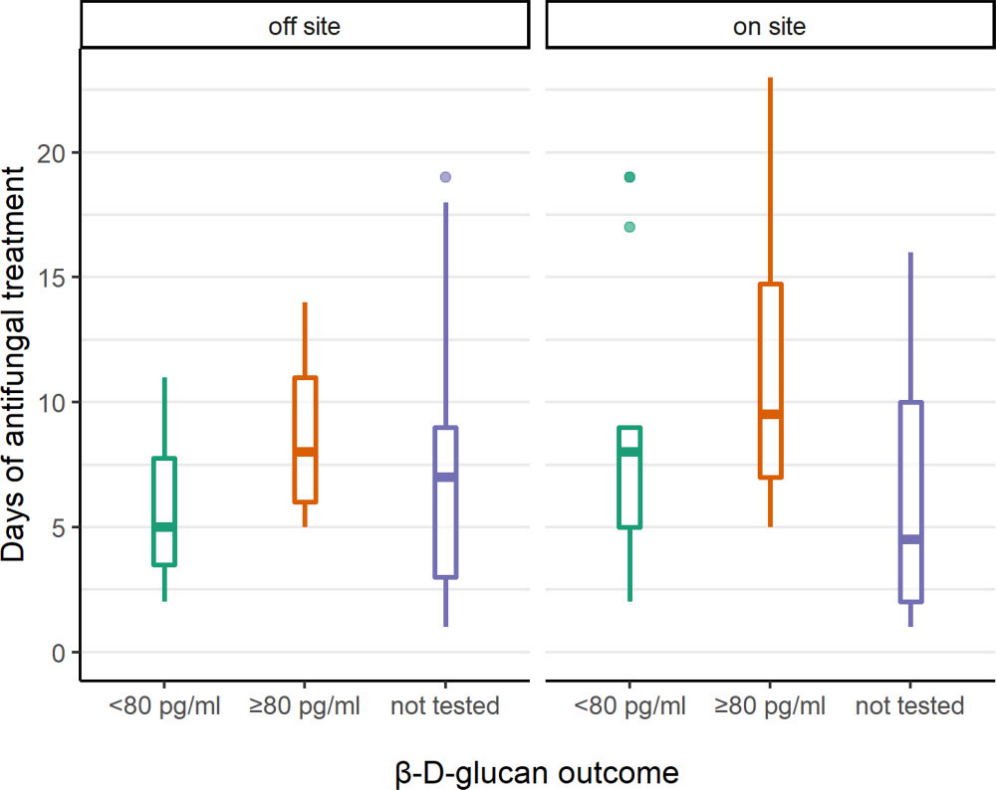
AFT duration by BDG outcome and testing location in patients with suspected but ultimately unproven invasive candidiasis. BDG outcome categories: BDG positive, ≥ 80 pg/mL; BDG negative <80 pg/mL; and not sent. On-site testing, duration of therapy by BDG outcome, median (IQR): BDG ≥ 80 pg/mL, 10 (7–15) days, *n *= 8; BDG <80 pg/mL, 8 (5–9) days, *n *= 17; BDG not sent, 5 (2–10) days, *n *= 26. Median BDG TAT: 1 day. Off-site testing, duration of therapy by BDG outcome, median (IQR): BDG ≥ 80 pg/mL, 8 (6–11) days, *n* = 9; BDG < 80 pg/mL, 5 (4–8) days, *n *= 18; BDG not sent, 7 (3–9) days, *n *= 72. Median BDG TAT: 11 days. For each box-and-whisker plot, the horizontal line represents the median, the upper and lower limits of the boxes the IQR, and the end of the whiskers 1.5 times the IQR. Outliers are shown as dots.

To explore the role of BDG in de-escalation, the relationship between BDG findings and antifungal duration in episodes prescribed for suspected but unconfirmed IC (*n *= 148) was analysed (Figure 2). Compared with episodes with a negative BDG result (< 80 pg/mL), episodes with a positive BDG (≥ 80 pg/mL) had on average 3.1 more DOTs (95% CI +0.1 to +6.2 DOTs, *P *= 0.05). There was no evidence that episodes in which BDG was never tested had different DOTs compared with a negative BDG (0.2 days, 95% CI −1.9 to +2.2 DOTs, *P *= 0.09). This was adjusted for testing location, as the BDG TAT was much shorter with on-site testing (KCH, median 1 day) compared with off-site testing (GSTT and SGH, median 11 days). Restricting the analysis to only on-site testing with readily available results did not reveal a statistically significant difference in duration, possibly due to the small sample size (compared with negative BDG, a positive BDG had +3.3 days, 95% CI −2.0 to +8.5 DOTs,  *P *=0.2; not sent −1.6 days, 95% CI −5.5 to +2.2 DOTs, *P *= 0.4).

Of all prescribing episodes where AFT was initiated for suspected IFI but where IFI was ultimately not proven (*n *= 186), 38% (*n *= 71) were discontinued and 4% (*n *= 8) de-escalated to a narrower-spectrum agent (echinocandin to fluconazole *n *= 7; amphotericin to mould-active azole *n *= 1) within 5 days. Hence, 58% (*n *= 107) of episodes were not stopped or de-escalated; an echinocandin, mould-active azole or amphotericin was prescribed for over 5 days in 39% (*n *= 73), and fluconazole only was given in the remaining 18% (*n *= 34).

## Discussion

Multiple drivers underlie decisions on starting or stopping antifungals in ICU patients, based on illness severity, assumptions about IFI frequency, at-risk populations, availability of on-site diagnostics and IFI knowledge amongst members of the multidisciplinary team (intensivists, microbiologists and pharmacists). The optimal approach to AFS is thus still being developed: our detailed descriptive evaluation of AFT use in general medical/surgical ICU at three large London healthcare organizations contributes to the limited evidence base by identifying key challenges for implementing AFS in the ICU setting.

Our evaluation found that 6.4% of all ICU admissions received AFT during their stay, reflecting other ICU-focused studies which report antifungal prescribing in 7.5%–9.2% of admissions in European and US ICUs.^25–28^ During the evaluation period, 1% (*n *= 50) of ICU admissions were admitted with or developed proven IFI, most commonly IC (92%), of which 62% was due to *C. albicans*. This reflects the findings of the much larger UK FIRE study (96 ICUs, 2009–11), which found 0.6% were admitted with or developed IC in the ICU (66% *C. albicans*).^29^ Furthermore, the incidence of IC (8.5 episodes per 1000 admissions), and specifically candidaemia (4.6 per 1000 admissions) is similar to rates reported in larger ICU-focused studies (7.07^3^ and 4.8–6.9^2–4,30^ per 1000 admissions for IC and candidaemia, respectively). Given the difficulties in attaining a definitive diagnosis of proven IC or IPA, we also classified probable and possible IFI cases, which accounted for a further 1% (*n *= 47) of admissions. Hence, in accordance with others’ findings, we found IFI was relatively uncommon in the general surgical and medical ICU, with proven, probable or possible IFI affecting ∼2% of patients during their ICU stay.

Echinocandins accounted for the greatest proportion of consumption (40%, 4.59 DOT/100 OBD), closely followed by fluconazole (38%, 4.31 DOT/100 OBD): together these two drug classes accounted for over three-quarters of all antifungal use. This is expected given echinocandins are recommended first-line therapy for IC in ICU and given the paucity of antifungal classes available.^31,32^ However the lack of diversity in prescribing illustrates why ICUs are fertile ground for fungal resistance emergence and hence the importance of stewardship in this setting. We further categorized consumption according to rationale to identify areas for focus in stewardship initiatives. The most common type of AFT prescribing was empirical (41% of all consumption), and prescribing was most frequently directed at an abdominal/GI source, accounting for 25% of all AFT use on ICU. Abdominal candidiasis is difficult to diagnose as blood cultures are insensitive and obtaining sterile intra-abdominal samples often not feasible. This diagnostic uncertainty leads to overprescribing, hence review of this subset of patients should be prioritized as a likely impactful area for the ICU AFS team.

When we considered the final diagnostic classification of all patients prescribed AFT for suspected or confirmed IFI (all empirical, pre-emptive, targeted prescribing); 23% ultimately had proven IFI and 22% probable or possible IFI. Thus in 55%, IFI was unlikely, illustrating potential for reduction in unnecessary prescribing. However, we also found that 52% with IC were not on AFT until microbiological diagnosis (i.e. yeast identified in blood/sterile site culture). Hence, empirical therapy is often not directed appropriately. Addressing this imbalance is a key aim of AFS in the ICU: to reduce the proportion of unnecessary AFT, while delivering timely and appropriate AFT for those who ultimately have IFI. Achieving the optimal balance is challenging as clinically differentiating IFI from other causes of deterioration in the ICU patient is difficult. A multi-pronged stewardship approach of diagnostic tool implementation, post-prescription review, provider education and surveillance is required to achieve this goal.

Non-culture-based tests (NCBTs) hold promise to overcome the limitations of culture-based fungal diagnostics by offering rapid results that can inform prescribing decision-making. Our evaluation highlighted issues around implementation that are applicable to many centres. Firstly, adequate TAT of NCBTs is essential if they are to have any impact on clinical decisions. TAT of BDG was significantly shorter when performed on-site, overcoming the challenges of packaging, transportation and manual upload of results from other laboratories with outsourced testing. Point-of-care, single-use diagnostics for *Aspergillus* GM and BDG are now available^33^ and could be used to provide ‘on-demand’ testing and reduce TAT.

In this evaluation, at the hospital with on-site BDG testing (TAT 1 day), in those with suspected but unproven IC, the median (IQR) duration of therapy was 8 (5–9) days with a negative BDG, compared with 10 (7–15) days with a positive BDG and 5 (2–10) days in those where the test was not sent. The difference in duration between the groups was not statistically significant, possibly due to the small sample size. However, further prospective exploration of the impact of BDG on prescribing practice is warranted. Confounding factors not measured in this evaluation that may influence duration of therapy, such as the degree of clinical suspicion of IC and clinical stability of the patient, must be considered. There is currently no guidance on management of ICU patients with a positive BDG but without culture-confirmed evidence of IC; the EORTC/MSGERC ICU working group has been unable to decide upon diagnostic criteria for probable and possible IC in ICU, due to a paucity of evidence on the diagnostic performance of NCBTs in non-neutropenic ICU patients.^34^ Longer therapy may be justified if BDG-guided AFT improves clinical outcomes; a trial using mortality endpoints in septic ICU patients with IC risk factors is ongoing.^35^ Single-centre RCTs^36,37^ and observational studies^38,39^ have suggested the negative predictive value of BDG could be used to reduce AFT duration in ICU, however others suggest its impact may be hindered by prolonged test TAT and the low positive predictive value for IC when used indiscriminately rather than targeted at high-risk patients.^40,41^ Given the effort and resources required to deliver timely NCBTs such as BDG in ICU, larger randomized-implementation trials are required to understand their impact on prescribing practice, clinical outcomes and their cost effectiveness.

Timely prescription review is a cornerstone of stewardship. Stopping (38%) or de-escalation (4%) of AFT within 5 days when suspected IFI was not proven was more commonly practised than in other studies, which report combined stopping and switching rates of only 20%–22%.^23,24^ This may reflect that all sites had an ICU microbiology ward round daily on weekdays incorporating post-prescription reviews of antimicrobials, such that most prescriptions (79%) were reviewed promptly at a median (IQR) of 1 (0–3) day. There is scope to improve the rate of post-prescription review, which provides an opportunity to assess microbiological results, optimize drug choice and dose and consider de-escalation. All sites have implemented processes to identify AFT prescriptions electronically. Improvement in advice documentation is required to increase the likelihood of advice acceptance. Building AFS into existing regular ICU/microbiology ward rounds allows timely post-prescription review and feedback and fosters the building of collaborative relationships and knowledge exchange, with mutual understanding of clinical context. While pre-prescription authorization of antifungals is another potential AFS strategy, the illness severity and out-of-hours nature of critical care may hamper its acceptability and application in this setting. To enhance the impact of reviews, the infection, pharmacy and ICU teams must develop specialist expertise in antifungal start–stop decisions; interpretation of fungal diagnostics and IFI management, drug–drug interactions and dosage adjustments in organ support, particularly for the complex ICU patient. Nourishing expertise in mycology requires teaching in this field to be better integrated in infection, pharmacy and ICU training.

While AFT consumption is a key stewardship metric, additional metrics are required that consider how appropriately antifungals are targeted in the ICU. This can include ultimate IFI diagnostic classification in those prescribed antifungals; time to AFT in proven infection; percentage of prescriptions reviewed and time to review; and percentage de-escalated at Day 5 of AFT. Additionally, consensus definitions of proven/probable/possible IFI in ICU patients are required to standardize stewardship guidance, management and research of IFI in this setting.^34^

Our study has several limitations. Evaluation and classification of final diagnostic classification was retrospective; not all patients had biomarker testing or *Candida* colonization screening, factors included in the definitions of probable/possible IFI, hence the number of patients in the probable/possible IFI categories may be underestimated. Undocumented (verbal—either in person or telephonic) microbiology advice and advice acceptance was not measured, which limits understanding of the impact of review on prescribing. Analysis of association of BDG outcome with duration of antifungal treatment is unadjusted; other confounding factors are likely to have influenced whether a test was performed and treatment duration. Moreover, this evaluation occurred prior to the COVID-19 pandemic, which has dramatically changed the case-mix in ICUs across the world, hampering both infection control and antimicrobial stewardship initiatives, with many COVID-19 ICU patients now receiving steroids and IL-6 inhibitors as part of their management. Together these factors will have changed the epidemiology of fungal infection in the ICU and likely driven an increase in empirical and targeted antifungal prescribing.

In summary, our in-depth evaluation of antifungal use in ICU has highlighted areas for focus in AFS. Optimizing AFT prescribing to achieve a better balance between early treatment for those with IFI while avoiding excessive empirical AFT use requires a multifaceted approach. Timely post-prescription review by specialist multidisciplinary teams that feed back and educate is central to this. Access to NCBTs, if ‘rapid’, offer potential to optimize prescribing decisions and stewardship of our limited antifungal armamentarium; however, an evidence gap must be addressed to understand what impact risk prescribing strategies that incorporate NCBTs in ICUs have upon AFT start and stop decision-making, allocation of resources and patient outcomes.

## Supplementary Material

dlac055_Supplementary_Data

## Transparency declarations

C.L. has received conference sponsorship from Gilead Sciences. D.W. has served as a board member for Camurus, GSK, Gilead and Menarini, and received speaker fees from Shionogi and bioMérieux. S.S. has received speaker fees from Gilead Sciences and Pfizer. T.B. reports research funding support from Gilead Sciences, Pfizer and Merck Sharp & Dohme, advisory board fees from Gilead Sciences and speaker fees from Gilead Sciences and Pfizer. C.H., A.F., J.E., A.M., P.W., A.G., P.H., J.B. and T.P. have nothing to declare.

## Supplementary data

Table S1 is available as Supplementary data at *JAC-AMR* Online.
