# Supplementary material for: A multisite evaluation of antifungal use in critical care: implications for antifungal stewardship
Source: JAC Antimicrob Resist. 2022 Jun 23;4(3):dlac055. doi: 10.1093/jacamr/dlac055 (PMC9217759; doi:10.1093/jacamr/dlac055)
Supplement: dlac055_Supplementary_Data [file dlac055_supplementary_data.docx]

**Supplementary data**

**Table S1. Criteria: Invasive Fungal Infection Diagnostic Classification**

| IFI Classification | Definition |
| --- | --- |
| Proven Invasive Candidiasis | Isolation of Candida sp. in (i) a blood culture or (ii) in a tissue/fluid sample taken from a normally sterile site obtained by sterile procedure (inc. drain specimens taken within ≤24 hours placement) with clinical or radiological signs of infection at that site. [22] |
| Probable IC | Meet all three criteria:  1. Evidence of infection: ≥48 hours antimicrobial therapy plus either one of:  (i) temperature ≥38 or <36, (ii) on inotropes or MAP <65 mmHg, (iii) WBC count ≥12 or <0.5 cells/µL)  2. Host risk factors: ≥ 2 of the following: mechanical ventilation, CVC line, TPN, ECMO, dialysis, major surgery, haematological malignancy, pancreatitis, steroids, immunosuppressive therapy.  3. Mycological evidence: Serum BDG level ≥80 pg/mL in two consecutive samples or BDG level ≥ 250 pg/mL in a single sample. |
| Possible IC | Meet all three criteria:  1. Evidence of infection: ≥48 hours antimicrobial therapy plus either one of:  (i) temperature ≥38 or <36, (ii) on inotropes or MAP <65 mmHg, (iii) WBC count ≥12 or <0.5 cells/µL)  2. Host risk factors: ≥ 2 of the following: mechanical ventilation, CVC line, TPN, ECMO, dialysis, major surgery, haematological malignancy, pancreatitis, steroids, immunosuppressive therapy.  3. Mycological evidence: *Candida* *sp*. colonization at ≥ 2 non-sterile sites. |
| Proven Invasive Mold infection | Isolation of a hyaline or pigmented mold by culture of a (i) specimen obtained by a sterile procedure from a normally sterile and clinically or radiologically abnormal site or (ii) a blood culture in the context of a compatible infectious disease process [22] |
| Probable IPA | Meets probable IA definition by either:   - 2019 EORTC/MSGERC criteria [22] - modified AspICU criteria [5]   2019 EORTC/MSGERC [22]: at least one host, clinical and mycologic criteria:  1. Host risk factors: ≥1 of the following: Neutropenia (< 500 neutrophils/mm^3^ for > 10 days), hematologic malignancy, allogenic stem cell transplant, solid organ transplant, prolonged corticosteroids, Treatment with recognized T-cell or B-cell immunosuppressants, Inherited severe immunodeficiency, grade III or IV graft versus host disease.  2. Clinical features: CT evidence of ≥1 of the following: dense, well-circumscribed lesions(s) with or without a halo sign, air crescent sign, cavity, wedge-shaped and segmental or lobar consolidation; tracheobronchitis visualized on bronchoscopy; CNS focal lesions or meningeal enhancement.  3. Mycological evidence: ≥ 1 of the following: Aspergillus recovered by culture from sputum, BAL, bronchial brush, or aspirate; positive direct examination showing hyphae; *Aspergillus* only: BAL fluid, serum or CSF galactomannan ≥1.0; serum ≥0.7 and BAL fluid ≥0.8; two consecutive positive Aspergillus PCR from plasma, serum, whole blood or BAL; or 1 in BAL and one in plasma, serum or whole blood.  Or meets the modified AspICU criteria [5] for invasive pulmonary aspergillosis:  1. Clinical criteria: ≥ 1 of the following: Fever refractory to ≥ 3 days of appropriate antibiotic therapy; Recrudescent fever after a period of defeverescence of ≥48 h while still on antibiotics and without other apparent cause; Dyspnoea; Haemoptysis; Pleural friction rub or chest pain; Worsening respiratory insufficiency in spite of appropriate antibiotic therapy and ventilatory support. 2. Radiological criteria: Any infiltrate on pulmonary imaging by chest X-ray or CT. 3. Mycological criteria: ≥ 1 of the following: Histopathology or direct microscopic evidence of dichotomous septate hyphae with positive culture for *Aspergillus* from tissue; *Aspergillus* culture from a BAL; A galactomannan optical index on BAL of ≥1; A galactomannan optical index on serum of ≥0·5. |
| Possible IPA | Meets 2019 EORTC/MSGERC criteria for possible IPA: an EORTC host factor and a clinical feature (as above), but no mycological evidence |
| IFI Unlikely | Does not meet any of above criteria |

Abbreviations: BAL, bronchoalveolar lavage; BDG, (1→3)-β-D*-*glucan*;* CNS, central nervous system; CT, computer tomography; CVC, central vascular catheter; ECMO, extracorporeal membranous oxygenation; EORTC, European Organisation for Research and Treatment of Cancer; IC, invasive candidiasis. IFI, invasive fungal infection; IPA, invasive pulmonary aspergillosis; MAP, mean arterial pressure; MSGERC, Mycoses Study Group Education and Research Consortium; TPN, total parental nutrition; WBC, white blood cell.
